# A Practical Narrative Review on the Role of Magnesium in Cancer Therapy

**DOI:** 10.3390/nu17142272

**Published:** 2025-07-09

**Authors:** Daniela Sambataro, Giuseppina Scandurra, Linda Scarpello, Vittorio Gebbia, Ligia J. Dominguez, Maria Rosaria Valerio

**Affiliations:** 1 Medical Oncology Unit, Ospedale Umberto I, 94100 Enna, Italy; daniela.sambataro@unikore.it; 2 Department of Medicine and Surgery, Kore University of Enna, 94100 Enna, Italy; giuseppa.scandurra@unikore.it (G.S.); ligia.dominguez@unikore.it (L.J.D.); 3 Medical Oncology Unit, Ospedale Cannizzaro, 95126 Catania, Italy; 4 Anesthesia and Intensive Care Unit, Ospedale Umberto I, 94100 Enna, Italy; scarpello.l@gmail.com; 5 Medical Oncology Unit, CdC Torina, 90145 Palermo, Italy; 6 Medical Oncology Unit, Policlinico, University of Palermo, 90127 Palermo, Italy; mariarosaria.valerio@unipa.it

**Keywords:** magnesium, carcinogenesis, cancer risk, hypomagnesemia, drug interactions

## Abstract

Magnesium (Mg^2+^) has gained oncologists’ attention due to its wide range of biological functions and frequent use as a complementary or integrative agent. This review outlines Mg’s actions, its complex role in carcinogenesis and tumor risk, and clinical issues. Mg^2+^ is essential in numerous biochemical processes, including adenosine triphosphate production, cellular signal transduction, DNA, RNA and protein synthesis, and bone formation. Pertinent full-text articles were thoroughly examined, and the most relevant ones were selected for inclusion in this review. There is conflicting scientific evidence about the relationship between Mg^2+^ changes and cancer risk, apart from colorectal cancer. Chronic Mg^2+^ deficiency leads to immune dysfunctions and enhanced baseline inflammation associated with oxidative stress related to various age-associated morbidities and cancer. On the other hand, Mg^2+^ deficiency is associated with drug or chemotherapy-related hypomagnesemia, postoperative pain, cachexia, opioid-induced constipation, normal tissue protection from radiation damage, and prevention of nephrotoxicity. A balanced diet usually provides sufficient Mg^2+^, but supplementation may be necessary in some clinical settings.

## 1. Introduction

In the last decades, there has been increasing interest in the role of magnesium (Mg^2+^) in many clinical scenarios, including aging and cancer [1,2,3]. Several diseases have been associated with Mg^2+^ deficits, such as cardiovascular and respiratory diseases; type 2 diabetes; respiratory airway constrictive syndromes; asthma; depression; stress-related, gastrointestinal, neurologic, and muscular diseases; liver cirrhosis; thyroid and parathyroid gland dysfunction; and cancer [4].

The role of Mg^2+^ in such a wide range of diseases is not surprising since Mg^2+^ plays an essential role in numerous cellular and physiological functions. It is involved in more than 600 enzymatic reactions involving ATP transfer, intermediary metabolism, potassium, and calcium transport, and acting as a co-factor in DNA, RNA, protein synthesis, and cellular proliferation [2,5]. Mg^2+^ is also essential for efficient energy production, oxidative phosphorylation, and glycolysis [5].

Despite Mg^2+^ being the second most abundant cation in the cell, its role in cellular physiology and pathology still needs to be elucidated. Mitochondrial RNA splicing protein 2 (MRS2), transient receptor potential cation channel subfamily M, member 6/7 (TRPM6/7), magnesium transporter 1 (MAGT1), solute carrier family 41 member 1 (SCL41A1), and cyclin and CBS domain divalent metal cation transport mediator (CNNM) proteins are among the Mg^2+^ transporters that physiologically regulate Mg^2+^ homeostasis [6]. Since Mg^2+^ is a crucial regulator of cell proliferation, Mg^2+^ transporters have a complex enabling relationship with cancer growth and metastatic dissemination [7]. The aberrant expression of Mg^2+^ transporters of the TRPM, CNNM, and SCL41 protein families is involved in tumor progression and may represent a potential therapeutic target [7]. Moreover, Mg^2+^ has complex relationships with aging, inflammation, and cancer [4]. Chronic Mg^2+^ insufficiency and aging are frequently associated with low-grade chronic inflammation and an excessive generation of free radicals, which may predispose individuals to cancer development [2,4].

Preclinical investigations on Mg^2+^ deficiency have found evidence of complex pro-inflammatory pathways in several cell types [8]. Fundamental studies on Mg^2+^ deficiency outline the roles of free radicals, cytokines, neuropeptides, endotoxins, antioxidants, and vascular permeability, along with related anti-inflammatory interventions. Mg^2+^ alterations are also associated with several events, such as genomic instability, telomere attrition, epigenetic alterations, loss of proteostasis, deregulated nutrient sensing, mitochondrial dysfunction, cellular senescence, stem cell exhaustion, altered intercellular communication, disabled autophagy, dysbiosis, and chronic inflammation [2,4]. Therefore, maintaining an optimal Mg^2+^ balance throughout life may help prevent oxidative stress and chronic conditions associated with aging.

Dietary Mg^2+^ and/or Mg^2+^ consumed in drinking water (generally more bioavailable than that contained in food) or alternative supplements should be considered for correcting deficits [9]. In addition to hard water, whole and unprocessed grains, seeds, chocolate, pecans, almonds, legumes, and green leafy vegetables are dietary optimal sources of Mg^2+^, recognized to be beneficial for human health. However, the daily dietary intake of Mg^2+^ is frequently below that recommended in Western countries [9]. 

As outlined below, Mg^2+^ and cancer have complex interrelations ranging from cancer risk to treatment-related issues. Therefore, the aim and the novelty of this narrative review is to provide oncologists with a practical overview of the role of Mg^2+^ in various cancer settings. The authors (DS, GS, LS, and MRV) conducted a literature review on MEDLINE, PubMed, EMBASE, and the Web of Science to report the relationship between Mg^2+^ and cancer selecting the works deemed relevant to the purpose of the narrative review, which were approved by other authors (LD, VG).

## 2. Magnesium and Carcinogenesis

Mg^2+^ plays a pivotal role in several biochemical reactions in the body, and its significance extends to cancer research and treatment. However, it is important to approach this issue knowing that scientific data are still emerging and that conclusive findings about the connection between Mg^2+^ and cancer are still pending.

The relationships between Mg^2+^, carcinogenesis, and cancer biology are remarkably complex and still need clarification, with conflicting results reported from many experimental, epidemiological, and clinical studies [10,11]. Mg^2+^ deficiency may result in both anti-neoplastic and pro-tumoral effects [11,12]. Preclinical studies showed that in vitro tumor cells tend to accumulate Mg^2+^, and high intracellular Mg^2+^ levels may confer a metabolic advantage, contributing to DNA alterations and promoting the acquisition of an immortal phenotype [11]. In Mg^2+^-deprived mice, low Mg^2+^ levels have paradoxical effects, both limiting and fostering carcinogenesis, since primary tumor growth inhibition may occur despite increased metastatic potential [11].

Nearly two decades ago, studies on preclinical models showed that Mg^2+^ depletion may inhibit solid tumor growth and paradoxically increase metastasis formation via enhanced tumor cell adhesion due to the altered expression of surface adhesion molecules [13]. Since then, Mg^2+^ has shown to influence the function of membrane ion channels in the tumor compared to normal tissues [13]. The increased expression and altered half-life of transporters/channels create a sustained high intracellular concentration of specific ions, e.g., calcium and chlorine that can activate proteolysis, promoting the invasive capacity of malignant cells by the degradation of the extracellular matrix, a necessary step for metastasis formation [14]. High calcium concentrations within tumor cells may also inhibit tumor immunity, and Mg^2+^, an essential intracellular and physiological calcium antagonist, may inhibit carcinogenesis [13,14].

Mg^2+^ can suppress carcinogenesis in a manner dependent on its intracellular concentration [8,9]. This phenomenon can be evidenced by the fact that Mg^2+^-deficient animals are at a higher risk of developing carcinomas, and most cancer patients are Mg^2+^-deficient. On the other hand, an optimal Mg^2+^ level reduces cancer risk by protecting cells from DNA damage by free radicals. Mg^2+^ reduces mutations to cells in vitro, and inactively dividing cells, like precancerous cells, are most vulnerable to low Mg^2+^ levels. Mg^2+^ also reduces tumorigenesis, slows its development, and decreases actin activity, cell motility, and the invasion of tumor cells [15,16,17,18]. Low Mg^2+^ levels are associated with a poor prognosis of cancer and aggravate systemic inflammation. Increasing extracellular magnesium concentration induces the apoptosis of cancer cells; however, Mg^2+^ supplementation does not cause a cancerous cell to die. It will speed up the growth of a cancerous cell as it needs energy to die.

Mg^2+^ and calcium ions control a diverse and essential range of cellular processes, such as gene transcription, cell proliferation, metabolism, neoplastic transformation, angiogenesis, metastatic potential, immune response, and treatment [17]. Mg^2+^ is a co-factor for numerous enzymes, including those involved in the body’s antioxidant defenses and DNA repair mechanisms. By supporting these processes, Mg^2+^ could theoretically reduce oxidative stress and genomic instability in cancer development. Mg^2+^ deficiency has been associated with inflammation and increased free radicals, where inflammatory mediators and free radicals could cause oxidative DNA damage and tumor formation [6,7]. In addition, Mg^2+^ is involved in mitochondrial function, apoptosis, and resistance to treatment. Alteration in Mg^2+^ channels’ expression and/or activity is frequent in cancer cells and human tumor tissues [7,18]. In vivo, Mg^2+^ deficiency and the consequent inflammation can trigger anti- and pro-tumor effects. Alterations in the expression of the TRPM7 epithelial Mg^2+^ channel are frequent in cancer cells and human tumor tissues. Mg^2+^ deficiency correlates with cell proliferation and/or migration [6,7]. TRPM7 may play an essential role due to its channeling function mediating Ca^2+^ and Mg^2+^ influx and kinase activity, likely regulating actomyosin contractility [7,18].

Mg^2+^ plays an essential role in energy production by acting as a co-factor for most glycolysis enzymes, the serine synthesis pathway, and the tricarboxylic acid cycle [19]. Glucose is the primary energy substrate for cancer cells, which can reprogram their metabolism to grow. Recent findings have shown that the dual-specificity phosphatases of regenerating liver (PRL-1, -2, and -3) play a role in oncogenesis and metabolic reprogramming mediated by Mg^2+^ homeostasis, shifting the energy source preference to glucose consumption and fueling the serine/glycine pathway and regulating the PI3 kinase/mammalian target of the rapamycin complex [19].

Overall, adequate Mg^2+^ levels are thought to help maintain normal cell function and could potentially perform a protective action against the development of certain cancers, although the clinical translation of the experimental data and the role of supplementation in the clinical settings (see below) remains unresolved.

## 3. Magnesium and Cancer Treatment

**Figure 1:**
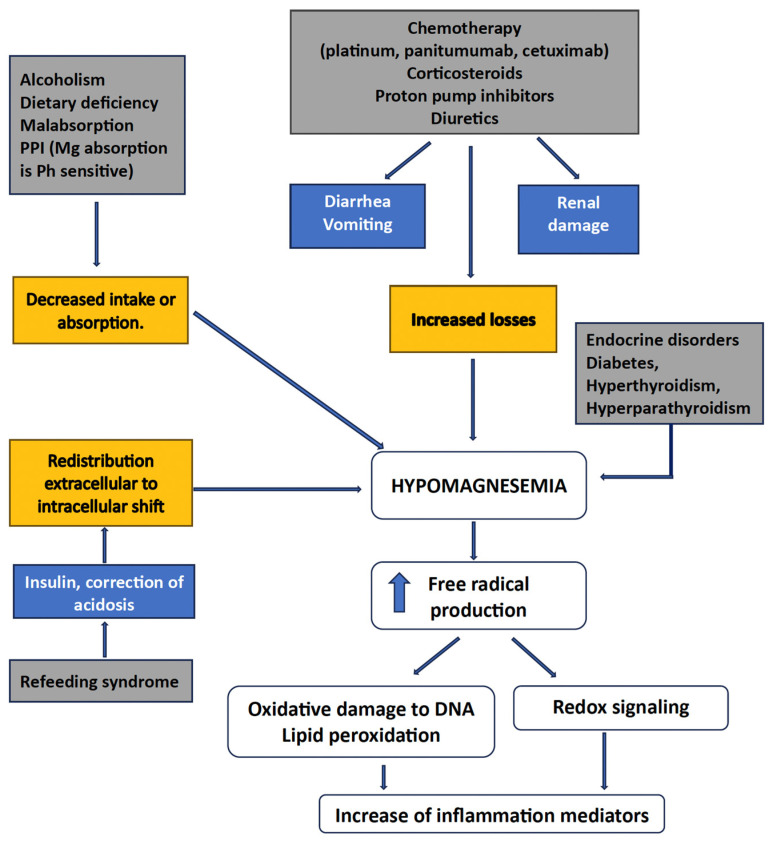
Interrelations between Mg^++^ and various cancer settings.

Mg^2+^’s role in cancer treatment is multifaceted and not entirely clear. Some anti-cancer agents can cause hypomagnesemia, which often necessitates Mg^2+^ supplementation to alleviate symptoms and support overall health during treatment [16]. However, whether Mg^2+^ supplementation directly contributes to cancer treatment efficacy or outcomes is still uncertain. Figure 1 shows the interrelations between hypomagnesemia and cancer-associated features.

### 3.1. Hypomagnesemia

Disorders of divalent ions are frequently observed in cancer patients, most notably hypomagnesemia, hypocalcemia, hypercalcemia, and hypophosphatemia [20]. These electrolyte imbalances may be related both to the side effects of anti-cancer therapy and the underlying malignancy as paraneoplastic syndromes. Electrolyte imbalances impact negatively on patients’ quality of life, leading to potentially life-threatening complications that may require hospitalization and usually carry a dismal prognosis [20,21]. Drugs linked to hypomagnesemia include antibiotics (e.g., aminoglycosides, amphotericin B), diuretics, anti-neoplastic drugs (platinum salts and anti-EGFR antibodies), calcineurin inhibitors, and proton pump inhibitors. The recognition, treatment, and prevention of hypomagnesemia in patients receiving high-risk anti-cancer agents are mandatory, as well as developing effective therapeutic and preventive strategies [22].

The causes of hypomagnesemia can be categorized according to the following pathophysiologic mechanism: decreased intake, transcellular shift, and gastrointestinal and renal losses [23,24]. Indeed, several causes of hypomagnesemia may coexist in a cancer patient, especially if elderly, as is frequently the case today, for example, a low dietary magnesium intake—typical of Western diets—increased urinary losses, and impaired intestinal absorption resulting from various pathological or iatrogenic conditions [2,4]. It is essential to remember that magnesium deficiency can have important implications due to the increased production of inflammatory markers, reactive oxygen species leading to DNA oxidative damage, and lipid peroxidation [2]. In addition, cancer patients may suffer from opportunistic infections and cardiovascular complications, therefore receiving drugs that can cause or exacerbate hypomagnesemia [20,24]. Moreover, several anti-cancer therapies are directly responsible for hypomagnesemia, including platinum-based chemotherapy, anti-EGFR monoclonal antibodies, human EGFR-2 target inhibitors, and calcineurin inhibitors [24]. Urinary indicators, such as the fractional excretion of Mg^2+^, can help etiology identification. 

The approach to managing hypomagnesemia varies based on its severity and underlying cause. Serum Mg^2+^ levels should be obtained before oncological treatments and routinely monitored throughout treatment. Nevertheless, Hsieh et al. conducted a systematic review and meta-analysis of retrospective and randomized trials comparing hypomagnesemia with normal Mg^2+^ levels in 1723 wild-type KRAS metastatic colorectal cancer (CRC) patients [25]. Patients with hypomagnesemia demonstrated better progression-free survival (hazard ratio [HR]: 0.64; 95% confidence interval [CI): 0.47–0.88), overall survival (HR 0.72; 95% CI: 0.53–0.92), and objective response rate (risk ratio [RR]: 1.81; 95% CI 1.30–2.52), confirmed for progression-free survival also in the subgroup analysis [25]. On a general rule, the management of hypomagnesaemia depends on the severity of Mg^2+^ depletion and the clinical setting.

### 3.2. Platinum Salts

Platinum salts are still the cornerstone of many chemotherapeutic regimens. The use of platinum salts, particularly cisplatin, may cause symptomatic hypomagnesemia and significant nephrotoxicity [26]. Several systematic reviews showed that fluid hydration and parenteral Mg^2+^ supplementation prevent cisplatin-induced hypomagnesemia and nephrotoxicity, although the best schedule is uncertain [27,28].

### 3.3. Anti-EGFR Antibodies

Hypomagnesemia is a recognized side effect of the anti-epidermal growth factor receptor (EGFR) antibodies cetuximab- or panitumumab-based regimens for metastatic CRC [29,30,31,32]. Moreover, these monoclonal antibodies are often delivered with oxaliplatin, another potential contributing cause. By preventing the EGF-dependent activation of TRPM6, the primary cation channel in charge of Mg^2+^ transcellular absorption in the kidney and intestine, these anti-EGFR drugs may result in hypomagnesemia, which may affect tumor response to cetuximab, according to limited observations [29]. Petrelli et al. conducted a systematic review and pooled analysis of randomized studies on the risk of anti-EGFR monoclonal antibody-related hypomagnesemia [33] reporting an incidence of 17% among patients treated with anti-EGFR agents, which was significantly increased compared with control patients (overall relative risk of 5.83; *p* < 0.00001). The risk is particularly elevated with panitumumab, which is also associated with a higher incidence of diarrhea and dehydration. However, hypomagnesemia does not seem to be linked to any severe complications.

### 3.4. Proton Pump Inhibitors

Proton pump inhibitors (PPIs) are reportedly potential risk factors for hypomagnesemia [34]. A multicenter study with a propensity score-matched analysis assessed the impact of PPIs on the risk of hypomagnesemia in 165 patients with metastatic CRC receiving panitumumab [34]. The incidence of grade 3–4 hypomagnesemia was significantly higher in the PPI group than in the control group, both before (20.0% vs. 8.0%, *p* = 0.026) and after propensity score matching (16.2% vs. 0%, *p* = 0.025). In the propensity score-matched cohort, the risk of grade 3–4 hypomagnesemia was doubled in the PPI group (OR 2.19; 95% CI 1.69–2.84; *p* = 0.025).

### 3.5. Dietary Mg^2+^ Supplementation

Adequate dietary Mg^2+^ may be protective against chemotherapy-associated hypomagnesemia. Liu et al. carried out a daily dietary replacement approach through Mg^2+^-rich foods to maintain adequate magnesium levels during platinum-based treatment for ovarian cancer [35]. Among 26 patients enrolled, those who were adherent to the diet (76%) had a significantly lower incidence of hypomagnesemia (19% versus 80%, *p* = 0.03) and less need for intravenous Mg^2+^ (6% versus 60%, *p* = 0.03) than those who were non-adherent.

### 3.6. Cardiotoxicity

Chemotherapy and newer targeted therapies may cause dangerous cardiovascular adverse events. The National Health and Nutrition Examination surveyed 1807 cancer survivors, followed-up for seven years. Overall, 51% of patients died of cancer and 33% of heart disease [36]. The potential cardiovascular toxicity of anti-cancer agents included QT prolongation syndrome, which is associated with potentially lethal cardiac arrhythmias. The treatment of such adverse events consists of an intravenous administration of Mg^2+^ sulfate and electrical cardioversion [37]. Mg^2+^-dependent TRPM7 plays a potential role in the cardiovascular toxicity of tyrosine kinase inhibitors during cancer treatment [38].

### 3.7. Nephrotoxicity and Nephroprotection from Chemotherapy

Nephrotoxicity is the primary dose-limiting toxicity of several anti-neoplastic agents, primarily platinum salts [39,40]. Various hydration regimens and supplementation protocols prevent cisplatin-induced kidney injury in clinical practice. However, evidence-based recommendations on specific hydration regimens are limited. A systematic review evaluated clinical studies that explored cisplatin-induced nephrotoxicity preventive strategies [39]. Four reported studies on Mg^2+^ supplementation demonstrated its role in providing nephroprotection. Besides hydration and forced diuresis with mannitol, Mg^2+^ supplementation (8–16 mEq) is beneficial and essential for all patients to prevent cisplatin-induced nephrotoxicity.

A second systematic review including 22 placebo-controlled trials extracted the number of patients, doses of cisplatin and protectant, and qualitative (acute kidney injury incidence) and quantitative (plasma creatinine, blood urea nitrogen, and creatinine clearance) indicators of renal function [40]. The results show significant variability in the nephroprotective capacity of various products evaluated. Of all the compounds tested, only Mg^2+^ sulfate and cystone were found to exert protective effects. A more recent systematic review comprising 11 studies fulfilling the research criteria showed that Mg^2+^ supplementation protected significantly against cisplatin-induced nephrotoxicity (OR = 0.22, 95% CI 0.14 to 0.35) [28]. However, further studies are needed to validate these findings.

A recent multicenter cohort study of 13,719 cancer patients who received prophylactic intravenous Mg^2+^ showed a lower risk of cisplatin-related acute renal injury as compared to those without supplementation [41]. Tanzawa et al. reported that early serum Mg^2+^ reduction in patients with advanced lung squamous cell carcinoma treated with chemo-immunotherapy was associated with a poor prognosis in terms of progression-free and overall survival, even after multivariate analyses [42]. However, both papers suggest caution in interpreting the data until randomized clinical trials will confirm these findings. A recent systematic review showed only weak evidence that Mg^2+^ supplementation may prevent the development or worsening of anti-EGFR antibody-related hypomagnesemia and reduce arrhythmia incidence [43]. On the other hand, Feng et al. recently reported a better outcome in cancer patients with elevated Mg^2+^ treated with anti-EFGR agents [44].

### 3.8. Radioprotection During Cancer Therapy

Radiotherapy may damage the tumor’s surrounding tissues, disrupting normal physiological functions. The patient’s quality of life may be drastically changed by symptoms including diarrhea, tenesmus, incontinence, and rectal bleeding, which are frequently brought on by this adverse impact. Patients may occasionally be more susceptible to micronutrient deficits and protein-calorie malnutrition. In addition, maintaining adequate levels of vitamin D, vitamin E, vitamin A, calcium, magnesium, thiamin, riboflavin, and niacin is required as part of a more comprehensive nutritional plan that includes energy (28–31 kcal/kg/day, using the Harris–Benedict formula adjusted for body weight in obese patients), protein (20–30%), fat (30–40%), and carbohydrates (40–50%). The patient’s age, nutritional state, and the existence of comorbidities must all be considered when determining their dietary needs [41,45].

### 3.9. Cachexia

The European Palliative Care Research Collaboration developed clinical guidelines for cancer patients suffering from cachexia [42]. Of the 21 publications included in the systematic literature research, only one study examined the use of Mg^2+^, which had no effect on weight loss. There is insufficient solid evidence to support using minerals, vitamins, proteins, or other supplements to treat cancer. No serious adverse effects have been reported with dietary supplementation [42,46].

### 3.10. Chemotherapy-Induced Peripheral Neuropathy

Platinum salts, taxanes, vinca alkaloids, and antimetabolites commonly induce peripheral neuropathy, which may significantly impair patients’ physical abilities and quality of life, often leading to dose reduction and/or discontinuation of chemotherapy treatments. Unfortunately, a systematic review of the potential use of nutraceuticals, including Mg^2+^, did not show significant evidence of clinical benefit to recommend supplements for the treatment or prophylaxis of peripheral neuropathy [47,48]. Managing chemotherapy-induced peripheral neuropathy remains a significant challenge, and further research is needed before recommending the use of supplements [49].

Loprinzi et al. reported a large trial that failed to show differences in the incidence of grade ≥2 neuropathy (National Cancer Institute Common Terminology Criteria for Adverse Events, NCI-CTC) between patients treated with Mg^2+^ infusions vs. controls (RR 0.81, 95% CI 0.60–1.11) [50]. A systematic review by Jordan et al., including five trials with 694 evaluable patients, failed to show a beneficial effect of Ca^2+^ and Mg^2+^ infusions to prevent oxaliplatin-induced peripheral neuropathy [47,51]. Efficacy endpoints were chronic neurotoxicity measured with NCI-CTC and the oxaliplatin-specific scale.

A review by Tofthagen et al. analyzed four studies exploring the relationship between Mg^2+^ levels and chemotherapy-induced peripheral neuropathy (CIPN) in patients with CRC treated with oxaliplatin [52]. Overall, there was a prevalence of hypomagnesemia in the range of 13.8–26% with significant differences (*p* = 0.05) between patients who developed neuropathy and those who did not. Pre-treatment hypomagnesemia was present in 80% of cases who developed grade 2–3 neuropathy, compared to only 20% of those who developed grade 0–1 neuropathy (*p* = 0.0001). A higher dietary Mg^2+^ intake was associated with a lower prevalence and less severe CIPN symptoms among CRC patients who received adjuvant chemotherapy with oxaliplatin [53]. However, further studies are needed to confirm the findings and to provide a solid basis for future recommendations directed toward the intake of magnesium before and during chemotherapy.

### 3.11. Cancer Pain

Mg^2+^ is frequently used daily to reduce pain intensity and analgesic consumption. The results are, however, only partially accepted. A review investigated randomized clinical trials on the effectiveness of Mg^2+^ treatment on pain and analgesics consumption in postoperative pain, renal pain, chronic pain, and neuropathic pain. The evidence for the efficacy of Mg^2+^ in reducing pain and analgesic consumption is modest globally. The literature has identified some gaps, especially in the methodology, rheumatic diseases, and cancer [54]. 

Surgically treated women with BC commonly suffer from neuropathic pain syndrome, which is a common yet debilitating complication that often decreases patients’ quality of life. Recently, emerging evidence has supported the therapeutic effect of Mg^2+^ administration on chronic pain. Ni et al. performed a randomized, double-masked, placebo-controlled clinical trial on 109 patients who received oral magnesium-L-threonate (*n* = 48) or placebo (*n* = 61) for 12 weeks [55]. The incidence of chronic pain was assessed at 3- and 6-month follow-up intervals. Nearly 31% of patients reported chronic pain after Mg^2+^ supplementation and 26% of the control group at the 6-month follow-up. Total scores of the short form of the Mc Gill Pain Questionnaire were significantly increased in the control group 6 months after surgical intervention (mean difference, 1.475; 95% CI, −2.730 to −0.2211; *p* = 0.002) but not in the Mg^2+^-treated group (mean difference, 1.250; 95% CI, −2.775 to 0.2748; *p* = NS). No significant differences were found between the two cohorts regarding questionnaire scores each time. Oral supplementation of magnesium-L-threonate did not effectively prevent the development of persistent pain in BC survivors nor provide sufficient pain relief over the placebo. However, the data show improved pain, mood, sleep disorder, or cognitive function after the 12-week Mg^2+^ supplementation. Patients treated with intravenous Mg^2+^ needed significantly fewer narcotics for pain control on postoperative days, even if recovery parameters, including maximal pain scores, postoperative mobilization, and length of hospital stay, did not significantly differ between the two groups [56]. Preemptive use of Mg^2+^SO_4_ reduces postoperative pain scores without affecting hemodynamic parameters during the induction and maintenance of general anesthesia [57].

### 3.12. Opioid-Induced Constipation

Constipation is a frequent gastrointestinal side effect of opioid treatment with a prevalence of up to 59%, usually managed with osmotic agents, stimulant laxatives, peripherally acting µ-opioid receptor antagonists, or naloxone [58]. International guidelines recommend standard laxatives such as macrogol/electrolytes and Mg^2+^ hydroxide to prevent opioid-induced constipation, although evidence from randomized controlled trials is weak. A systematic review and meta-analysis of twenty-two trials analyzed the scientific evidence on pharmacological strategies for the prevention and treatment of opioid-induced constipation in cancer patients. Overall, the data failed to show apparent differences in the efficacy of the laxatives [59]. Only one cohort study showed a significant benefit of Mg^2+^ oxide compared with no laxatives. One randomized trial found a significant benefit for naldemedine compared with Mg^2+^ oxide. A large Japanese retrospective study used a nationwide hospital claims database to compare naldemedine and Mg^2+^ oxide as first-line laxatives in cancer patients on long-term opioid therapy, with ≥6 months of post-opioid follow-up [60]. After propensity score matching, the incidence of death was not adjusted enough and was significantly higher in the naldemedine arm (1717 patients) than in the Mg^2+^ oxide arm (544) in the non-early group, but comparable in the early group. The incidence of addition, change, or dose increase was significantly higher in the naldemedine arm than in the Mg^2+^ oxide arm of the early-prescription group (HR 1.08; 95% CI 1.00–1.17; *p* = 0.0402). An open-label, randomized, multicenter study on 330 patients with opioids for pain management to examine if Mg^2+^ hydroxide is non-inferior to macrogol/electrolytes in the prevention of opioid-induced constipation is ongoing [61]. Overall, magnesium oxide and naldemedine are most likely effective for the prevention of opioid-induced constipation in cancer patients, even if further studies are needed before establishing sound recommendations for clinical practice.

### 3.13. Other Drug Interactions

Mg^2+^ salts retain water in the intestine and act as an osmotic laxative. They may reduce the bioavailability of concomitantly administered drugs by forming insoluble chelate complexes with those drugs [62]. Besides calcium, bisphosphonates and high-dose antiviral agents may affect Mg^2+^ levels [63,64]. Mg^2+^ may reduce the absorption of the antifungal itraconazole and several antibiotics, including fluoroquinolones, tetracyclines, and nitrofurantoin [65,66,67].

## 4. Magnesium and Cancer Risk

Consumption of Mg^2+^ from dietary sources may be beneficial in reducing all-cause and cancer mortality and thus have practical importance for public health. Table 1 synthetizes the main studies on the association between Mg^2+^ and cancer risk per type of disease. Some epidemiological studies have suggested a non-causative correlation between higher dietary Mg^2+^ intake and a lower risk of some malignancies, such as CRC [68]. Mg^2+^ levels correlated with an increased risk of breast cancer. In endometrial and esophageal cancers, no risk association with Mg^2+^ was found, while a weak or stronger correlation was observed in lung and thyroid cancers. Higher levels of serum Mg^2+^ significantly associated with a decreased risk of HCC among patients with nonalcoholic fatty liver disease. All these studies often rely on dietary surveys and need more accuracy. Despite these limitations, the data hint at Mg^2+^’s potentially beneficial role in cancer prevention. 

Bagheri et al. carried out a meta-analysis of nineteen prospective studies, including 11,408 cancer-related deaths, to examine the association of total, supplemental, and dietary Mg^2+^ intake with the risk of all-cause, cancer, and cardiovascular disease mortality, and identify the dose–response relations involved in these associations [69]. Dietary Mg^2+^ intake was associated with a lower risk of all-cause [*p* < 0.001] and cancer mortality (*p* = 0.027). However, the analysis did not show any statistically significant associations between supplemental and total Mg^2+^ intake with cancer and risks of all-cause mortality. However, linear dose–response meta-analysis indicated that each additional intake of 100 mg/d of dietary Mg^2+^ was associated with a 6% and 5% reduced risk of all-cause and cancer mortality, respectively [69]. 

In 2022, the US Preventive Services Task Force reported a large, pooled analysis to establish the role of vitamins and mineral supplements for primary cancer prevention, including eighty-four studies and 739,803 patients [70]. While multivitamin use was significantly associated with a lower incidence of any cancer in 4 clinical randomized trials comprising 48,859 cases (OR 0.93; 95% CI 0.87–0.99), the evidence for the benefit of other supplements was equivocal, minimal, or absent, or even dangerous. Beta carotene was significantly associated with an increased risk of lung cancer (OR 1.20, 95% CI 1.01–1.42) and cardiovascular mortality (OR 1.10; 95% CI 1.02–1.19) [71].

### 4.1. Colorectal Cancer

Epidemiologic studies have suggested that Mg^2+^ intake may be correlated to a decreased risk of CRC, but the findings have been inconsistent [72].

A study of 412,000 subjects from 12 prospective studies followed-up for 8–14 years showed an inverse relationship between Mg^2+^ intake and risk of CRC in men but not women [73]. In the US, high Mg^2+^ levels in drinking water and living in the highest quartile have a significantly lower risk of all cancers in both genders.

Swedish researchers carried out a population-based prospective trial on a cohort of 61,433 women showing an inverse association of magnesium intake with the risk of colorectal cancer (*p* for trend = 0.006) suggesting that a high Mg^2+^ intake may reduce the occurrence of colorectal cancer in females [74]. Cohort-study meta-analyses revealed that an elevated dietary Mg^2+^ intake was adversely linked to the risk of CRC, with HRs of 0.76 (95% CI 0.72, 0.80) and 0.80 (95% CI 0.73, 0.87). High dietary intakes of calcium, Mg^2+^, and potassium were found to be negatively associated with the incidence of CRC in a meta-analysis of case–control studies. The corresponding ORs were 0.78 (95% CI 0.63, 0.98), 0.97 (95% CI 0.74, 1.21), and 0.36 (95% CI 0.32, 0.40) [72].

Chen et al. reported a meta-analysis of eight prospective studies comprising 338,979 participants and 8000 CRC cases, showing that a higher Mg^2+^ intake may be associated with a modest decrease in CRC risk, particularly colon cancer [73]. The summary relative risk (RR) for the highest vs. lowest category of Mg^2+^ intake for CRC was 0.89 (95% CI, 0.79–1.00), with little evidence of heterogeneity. For colon and rectal cancers, the pooled RR was 0.81 (95% CI, 0.70–0.93) and 0.94 (95% CI, 0.72–1.24), respectively. In the dose–response analyses, the summary RRs for an increment in Mg^2+^ intake of 50 mg/day for CRC, colon, and rectal cancers were, respectively, 0.95 (95% CI, 0.89–1.00), 0.93 (95% CI, 0.88–0.99), and 0.93 (95% CI, 0.83–1.04), and there was some evidence of heterogeneity; omitting one study that substantially contributed to the heterogeneity yielded generally similar results, but with low heterogeneity.

The evidence from 80 meta-analyses of interventional and observational trials of CRC prevention utilizing drugs, vitamins, supplements, and dietary variables was assessed in another review [69,75]. Mg^2+^, aspirin, non-steroidal anti-inflammatory drugs, folate, and a high consumption of fruits and vegetables, fiber, and dairy products were associated with a decreased incidence of CRC [69,75]. The relationship between all prescriptions of nearly 400 drugs other than anti-neoplastic ones and the mortality of CRC patients was analyzed in 2618 cases registered in the Korean National Health Insurance Service National Sample Cohort database, controlling for multiple comparisons with the false discovery rate [70,76]. The results suggest that Mg^2+^ may have a detrimental effect on CRC prognosis [76].

### 4.2. Esophageal Cancer

A large, nested case–control study employing a self-reported dietary assessment showed no associations between copper and Mg^2+^, and the risk of esophageal cancer [71,77]. The median serum concentration of Mg^2+^ was 41.7 ppm in patients with esophageal cancer and 50.0 ppm in controls, with no associations between magnesium and esophageal cancer risk in either the tertile models or the continuous estimate (magnesium: OR T3 vs. T1 = 0.75, 95% CI = 0.32–1.78) [77].

### 4.3. Lung Cancer

Whether serum Mg^2+^ levels are lower in lung cancer patients than healthy controls is still controversial. A meta-analysis by Song et al. including eleven articles (707 lung cancer cases and 7595 healthy controls) suggests that the relationship between serum Mg^2+^ levels and lung cancer is not relevant [78], with similar serum Mg^2+^ levels in patients and controls, and significant heterogeneity (I^2^ = 99.6%, *p* < 0.001). Zhai et al. reviewed the possible role of magnesium in reducing lung cancer risk since this cation has shown protective effects against lung function loss [79].

### 4.4. Thyroid Cancer

A systematic review of 11 epidemiological studies examining the association between dietary mineral supplements, including Mg^2+^, and thyroid cancer risk found largely inconsistent results across studies [80]. There is conflicting evidence regarding the relationship between thyroid cancer and serum levels of selenium, copper, and Mg^2+^. A meta-analysis involving 1291 participants reported a strong correlation between thyroid cancer and serum cation levels, revealing that individuals with thyroid cancer had elevated copper levels but reduced selenium and Mg^2+^ levels compared to healthy controls [81]. Nonetheless, the subgroup analysis revealed a noteworthy ethnic variation in copper and selenium levels. To better understand the causal relationship between selenium, copper, and Mg^2+^ and thyroid cancer across different populations and regions, a multicenter, trans-regional study is needed to confirm these findings [81].

### 4.5. Breast Cancer (BC)

Viable BC cells increase the expression of Mg^2+^ transport channels, which raises the intracellular concentration of the mineral [82,83]. This contributes to tumor growth by increasing energy demand [76,77]. These data, however, need confirmation. Kim et al. investigated the causal effects of micronutrient levels on cancer by applying the Mendelian randomization method, using single-nucleotide polymorphisms linked to micronutrient levels as instrumental variables [84]. The results favor an increased risk of BC correlated with Mg^2+^ levels (OR = 1.281; *p* < 0.0001).

A review on the alterations in Mg^2+^ homeostasis in BC suggest a possible interference with BC progression, even if the existing data are scarce and inconsistent [83]. A case–control study of 1050 patients and 1229 controls showed that a higher Mg^2+^ intake was associated with a lower BC risk possibly via its effect on inflammatory markers C-reactive protein (CRP) and interleukin-6 (IL-6). A positive association was found between the CRP level and BC risk (adjusted OR = 1.43) while IL-6 showed no association [85]. However, in a cohort of 1170 women with BC, a higher dietary intake of Mg^2+^ was inversely associated with all-cause mortality primarily among postmenopausal women with a high Ca:Mg intake ratio. No clear associations were observed between calcium intake and prognosis [86]. The results suggest that Mg^2+^ intake alone may improve BC overall survival. In women with BC, reduced serum Mg^2+^ concentrations may impair the antioxidant defense systems involved in the carcinogenesis process as evaluated by superoxide dismutase activity and its relationship with oxidative stress markers. Bezerra et al. demonstrated that women with BC had lower mean dietary Mg^2+^ and erythrocyte levels than the control group (*p* < 0.0001) and were insufficient by reference standards [87]. There was a significant difference in urinary excretion between the groups, with levels being elevated (*p* < 0.0001).

### 4.6. Cervical Cancer

The National Health and Nutrition Examination Survey study explored dietary intake correlations among 215 women affected by cervical cancer and 860 without cancer [88]. The research implemented the univariate analysis and the least absolute shrinkage and selection operator regression to estimate the association of 29 variables with cervical cancer, subsequently identifying the most pertinent variables linked to cervical cancer. Among the six covariates examined (age, race, fiber, Mg^2+^, caffeine, and vitamin C), Mg^2+^ was linked to cervical cancer in univariate analyses (*p* < 0.05).

### 4.7. Endometrial Cancer

Obesity and endometrial cancer risk are strongly associated. Therefore, dietary habits may play an essential role in developing this cancer. Wang et al. conducted a study using two-sample mendelian randomization to explore the effects of circulating levels of 15 micronutrients, including Mg^2+,^ as well as corrected relative macronutrient intake (protein, carbohydrate, sugar, and fat) on risks of endometrial cancer and its subtypes (endometrioid and non-endometrioid histology) [89]. Vitamin C levels predicted by genetics were found to be highly correlated with the risk of endometrial cancer. There was some indication that the risk of endometrial cancer was influenced by the relative intake of macronutrients (fat, sugar, and carbohydrates) as predicted by genetics. No correlation with Mg^2+^ or other significant associations were observed.

### 4.8. Hepatocellular Carcinoma (HCC)

Mg^2+^ deficiency is commonly associated with liver diseases, and Mg^2+^ supplementation can improve liver function. Mg^2+^ deficiency may result from low dietary intake, increased urinary secretion, hypoalbuminemia, or hormone inactivation. Low Mg^2+^ content in serum and liver tissue may foster disease progression due to disruption in mitochondrial function, defective protein kinase C translocation, inflammatory responses, oxidative stress, or metabolic disorders [90]. In preclinical models, a high expression of protein phosphatase Mg^2+^-dependent 1H (PPM1H) mRNA and protein correlated with better prognosis. PPM1H inhibited the proliferation, migration, and invasion of hepatoma cell, and inhibited induced HCC nodule formation [91].

Epidemiological data on Mg^2+^ level and its relation to HCC are occasional. Nonalcoholic fatty liver disease is a major contributive factor to the increasing incidence of HCC. Yu et al. explored the associations between serum Mg^2+^ levels and the risk of HCC among 26,053 patients with nonalcoholic fatty liver disease [92]. Overall, 395 patients developed HCC after the first measurement of serum Mg^2+^. Patients with nonalcoholic fatty liver disease who developed hepatocellular carcinoma (HCC) had significantly lower average serum Mg^2+^ levels (0.769 ± 0.131 mmol/L) compared to those who remained cancer-free (0.789 ± 0.125 mmol/L; *p* = 0.003). This suggests that higher serum Mg^2+^ levels are significantly associated with a reduced risk of HCC in this patient population. A retrospective study showed that serum levels of Mg^2+^ were significantly lower in cirrhotic patients with HCC than in cancer-free ones on multivariate logistic regression [93].

**Table 1:** Mg^2+^ effects on risk across cancer types.

Clinical Setting	Main Findings	Study Type	References
Breast cancer	Increased risk of breast cancer correlated with Mg^2+^ levels	Randomized mendelian	Kim et al., 2023 [84]
	Higher dietary intake of Mg^2+^ was inversely associated with risk of all-cause mortality primarily among postmenopausal women	Retrospective	Tao et al., 2015 [86]
	BC patients have lower mean dietary Mg^2+^ and erythrocyte levels than the control group; Mg^2+^ correlated to oxidative stress	Case–control	Bezerra et al., 2021 [87]
Cervical cancer	Mg^2+^ was linked to cervical cancer in univariate analyses	Survey with Lasso regression	Xu et al., 2023 [88]
Colorectal cancer	Mg^2+^ intake correlated to decreased risk		Meng et al., 2019 [72]
	Inverse relationship between Mg^2+^ intake and risk of CRC in men but not women	Meta-analysis	Chen et al., 2012 [73]
	High Mg2+ intake may reduce the occurrence of colorectal cancer in females	Prospective cohort	Larsson et al., 2005 [74]
	Mg^2+^ associated with decreased risk of CRC	Review	Chapelle al., 2020 [75]
	Mg^2+^ supplementation associated with detrimental prognosis of CRC	Retrospective	Woo et al., 2023 [76]
Endometrial cancer	No association between Mg^2+^ or endometrial cancer	Prospective randomized	Wang et al., 2023 [89]
Esophageal cancer	No association of Mg^2+^ with risk of esophageal cancer	Nested case–control study	Hashemian et al., 2023 [77]
Hepatocellular carcinoma	Higher levels of serum Mg^2+^ significantly associated with decreased risk of HCC among patients with nonalcoholic fatty liver disease	Retrospective	Yu et al., 2023 [92]
	Low Mg^2+^ is associated with cancer cells in cirrhotic patients	Retrospective	Parrisse et al., 2021 [93]
Lung cancer	The relationship between serum Mg^2+^ levels and lung cancer is non-significant	Meta-analysis	Song et al., 2018 [78]
Thyroid cancer	Non-consistent results	Review and meta-analysis	Shen et al., 2015 [81]
	Low levels of Mg^2+^ associated with thyroid cancer	Review	Zhang et al., 2013 [80]

## 5. Therapeutic Perspectives

Understanding tumor biology and progressing studies on ion transporters provide new insights into the theoretical possibility of Mg^2+^ being used in cancer treatment by adding to or depleting these ion levels in specifically targeted tissues [83,84,94,95]. Given its role in ion competition, these findings lead to questions about the possibility of Mg^2+^ being a tumor suppressor and protector of host tissue. Mg^2+^ nanoclusters may enhance palbociclib activity [83,93]. Co-delivery of palbociclib with ultra-small magnesium nanoclusters was found to be more effective than free palbociclib at inhibiting cell growth in cytotoxicity assays conducted on estrogen receptor and folate receptor-positive breast cancer cells. Mg^2+^-based compounds may represent a new therapeutic option in the future. A series of novel, mixed transition metal–Mg^2+^ tartrate complexes were screened in vitro for their anti-cancer activity against human BC cell lines, showing antiproliferative activity and cell migration inhibition [95]. A double-blind, placebo-controlled clinical study failed to show the activity of Mg^2+^ in reducing hot flashes in patients with BC [96].

## 6. Discussion

The role of Mg^2+^ in cancer is a complex and intriguing area of study, with experimental and epidemiological evidence presenting a conflicting picture [83,97]. On the one hand, its involvement in cellular metabolism and maintaining genetic stability, regulation of cell proliferation, and protection against insulin resistance, oxidative stress, and systemic inflammation are considered cancer-preventive attributes [85,86,98,99]. On the other hand, hypomagnesemia, as a side effect of some cancer treatments, may produce an inhibitory effect on tumor growth and neo-angiogenesis. In fact, it has been shown that Mg^2+^ levels increase in both in vivo and in vitro tumors, suggesting its multiple and possibly dichotomous roles in cancer [18,87,100]. The preclinical and clinical research have shown low Mg^2+^ having both pro- and anti-tumor effects, including facilitating tumor implantation at metastatic sites and inhibiting tumor growth at its originating site. The controlled and coordinated variations in intracellular Mg^2+^ are drastically disrupted in several cell types by neoplastic transformation, which provides selective advantages to the cells. Moreover, this scenario is further complicated by inflammatory responses to hypomagnesemia even if Mg^2+^ immunomodulatory function regulates NF-kB activation and cytokine production and limits systemic inflammation, such as C-reactive protein and endothelial dysfunction [101,102].

This paradox highlights the dualistic nature of magnesium in oncological contexts. While sufficient Mg^2+^ levels support DNA repair mechanisms and immunologic surveillance—both critical in preventing malignant transformation—some studies suggest that reducing Mg^2+^ availability can suppress cancer cell proliferation by interfering with angiogenesis and altering the tumor microenvironment. Furthermore, magnesium’s role in modulating signaling pathways such as PI3K/Akt and NF-κB adds another layer of complexity, as these pathways can either promote or inhibit tumor progression depending on the context.

Moreover, patient-specific factors, such as genetic background, comorbidities (e.g., metabolic syndrome, and diabetes), menopausal status, and dietary patterns, may influence how magnesium status affects cancer risk and outcomes. As such, the therapeutic implications of magnesium in cancer remain ambiguous: while supplementation may be beneficial for cancer prevention or mitigating treatment side effects, in certain clinical settings, controlled magnesium depletion might enhance anti-tumor efficacy.

Future research should aim to clarify the context-dependent effects of magnesium by incorporating longitudinal data, stratifying patients by magnesium status and cancer type, and exploring molecular mechanisms in preclinical models. This nuanced approach could help determine whether magnesium acts more as a friend or a foe in the fight against cancer, or perhaps both, depending on the circumstances.

The Nordic Nutrition Recommendations suggested a recommended intake based on balance studies [103]. However, the average requirement still needs to be set. Functional indicators of magnesium status still need to be improved. A scoping review reveals new research on Mg^2+^ intake’s beneficial effect on several health outcomes (cardiovascular disease, diabetes, and some cancers). A causal association is suggested based on meta-analyses of cohort and randomized controlled trials. However, due to the limitations of these study designs, it is not possible to establish an optimal intake, and new balance studies are still lacking [103].

Applying the knowledge gained on Mg^2+^ from the experimental models to real-world situations is challenging [91]. While controlled laboratory settings provide valuable insights into magnesium’s molecular mechanisms—such as its roles in DNA repair, oxidative stress reduction, and immune regulation—these findings do not always translate seamlessly to human populations. The epidemiological research suggests that magnesium deficiency is associated with an increased risk of certain malignancies, including colorectal, pancreatic, and liver cancers. However, establishing a clear causal relationship remains difficult due to confounding factors such as diet, lifestyle, comorbid conditions, and genetic variability.

The issue is further complicated by the observation that magnesium homeostasis is often disrupted in cancer patients. Factors such as altered absorption, renal wasting, and the effects of chemotherapy or targeted therapies can all contribute to hypomagnesemia, which in turn may impact both tumor progression and patient outcomes. Moreover, some cancer treatments intentionally induce magnesium loss, raising questions about whether this effect is detrimental or potentially therapeutic in certain contexts.

This complexity underscores the urgent need for more translational and clinical research to better understand magnesium’s role across the cancer continuum—from risk and prevention to treatment response and survivorship. Longitudinal studies randomized controlled trials, and mechanistic investigations in diverse patient populations are essential to elucidate whether magnesium supplementation could serve as a viable preventive or adjunctive therapy, or whether magnesium modulation might be more nuanced and cancer-specific.

Ultimately, bridging the gap between bench and bedside will require a multidisciplinary approach, integrating the findings from basic science, nutrition, oncology, and personalized medicine. Only then can the full potential of magnesium as a modifiable factor in cancer prevention and care be realized [91,104].

Despite the evidence suggesting the potential protective effect of Mg^2+^ against cancer and its importance in managing treatment-related side effects, the current consensus is that there is insufficient data to recommend Mg^2+^ supplements specifically for cancer prevention or treatment [5], whether or not monitoring and/or supplementing Mg^2+^ to cancer patients depends on individual clinical situations [20,24]. Unless a healthcare professional advises supplementation, the best approach is to maintain adequate Mg^2+^ levels through a balanced diet rich in Mg^2+^-containing foods, such as nuts, seeds, whole grains, legumes, and leafy green vegetables. In other clinical settings, such as treatment with platinum-based chemotherapeutic agents (e.g., cisplatin or carboplatin) or anti-EGFR therapies, as well as in cases of clinically significant hypomagnesemia, it is strongly advisable to closely monitor serum Mg^2+^ levels. These therapies are well-documented to induce magnesium wasting through renal loss, which can lead to symptomatic hypomagnesemia characterized by neuromuscular disturbances, cardiac arrhythmias, and fatigue—potentially compromising the patient’s overall treatment tolerance and quality of life.

Regular monitoring is essential not only to prevent acute symptoms but also to maintain optimal physiological conditions that support immune function, electrolyte balance, and cardiovascular stability during cancer therapy. In particular, hypomagnesemia has been associated with worse outcomes in patients receiving anti-EGFR agents, as it may reflect an increased pharmacodynamic effect of the drug, possibly predicting both therapeutic response and increased toxicity.

Furthermore, prolonged magnesium deficiency may exacerbate treatment-related side effects, such as nephrotoxicity in platinum-based therapies or diarrhea in EGFR-inhibitor regimens. Timely detection through routine monitoring allows for early intervention—whether through oral or intravenous magnesium supplementation—thus minimizing treatment disruptions and improving patient safety.

Given the clinical implications, incorporating a magnesium status assessment into standard oncologic care protocols, especially in high-risk treatment regimens, represents a practical and cost-effective strategy to enhance patient management and outcomes. Future research should aim to define evidence-based guidelines for magnesium monitoring frequency, thresholds for supplementation, and its potential role as a biomarker for treatment efficacy or toxicity risk.

## 7. Conclusions

Researchers are actively investigating the relationship between Mg^2+^ and cancer to gain a deeper understanding of how nutritional factors affect cancer risk, progression, and treatment outcomes. This information could assist clinicians in determining whether to adjust the intake of specific micronutrients, especially in high-risk populations without existing nutritional deficiencies. Additionally, it may inform the design of future clinical trials. Future studies may offer clearer insights into the potential role of Mg^2+^ in cancer prevention and treatment. In conclusion, maintaining adequate Mg^2+^ levels supports overall health and could influence cancer risk and therapy outcomes. However, more research is necessary to fully understand its role and effectiveness in these areas.

## Abbreviations

The following abbreviations are used in this manuscript:

Mg^2+^	Magnesium^2+^
CRC	Colorectal carcinoma
EGFR	Epidermal growth factor receptor
HCC	Hepatocellular carcinoma
